# First-line therapy with sunitinib in advanced renal cell carcinoma: interpretation of the overall survival data from asco 2008

**DOI:** 10.3747/co.v16i0.405

**Published:** 2009-05

**Authors:** L. Wood

**Keywords:** Sunitinib, renal cell carcinoma, overall survival

## Abstract

Sunitinib is now a standard first-line therapy for metastatic clear-cell kidney cancer. This paper focuses on interpretation of the overall survival data presented at the 2008 annual meeting of the American Society of Clinical Oncology from the pivotal phase iii trial comparing sunitinib with interferon in the first-line setting. The previously published progression-free survival and response rate data from that study are also summarized.

## 1. INTRODUCTION

Sunitinib is an oral multi-targeted tyrosine kinase inhibitor of vascular endothelial growth factor (vegf) receptor and platelet-derived growth factor receptor that has become a new standard of care in the management of metastatic renal cell carcinoma (rcc). It was at the 2006 annual meeting of the American Society of Clinical oncology (asco) that Motzer *et al.* first presented the results of a pivotal multicentre phase iii trial in metastatic RCC that compared sunitinib with interferon alfa. The data were subsequently published in January 2007 1,2.

This study by Motzer and colleagues randomized previously untreated patients with metastatic clear-cell carcinoma to either oral sunitinib (50 mg once daily for 4 weeks, followed by a 2-week break) or subcutaneous interferon alfa (9×10^6^ IU 3 times weekly). The primary endpoint was progression free-survival (pfs), and secondary endpoints included overall survival (os), Response Evaluation Criteria in Solid Tumors objective response rates, patient-reported outcomes, and safety.

From a statistical viewpoint, the study was designed to have 90% power to detect a 35% improvement in pfs to 6.2 months from 4.7 months, using a two-sided *p* value of 0.05. In terms of os, the study was designed to have 85% power to detect a 35% improvement in os to 17 months from 13 months. It was estimated that 690 patients would need to be enrolled. Pre-specified plans were in place to analyze the survival data by Eastern Cooperative Oncology Group (ecog) performance status (ps), lactate dehydrogenase, and nephrectomy status, using unstratified and stratified log-rank tests, the Wilcoxon test, and the Cox model. The log-rank test is an appropriate test where the ratio of death rates between two treatment arms is constant over time. The Wilcoxon test is an appropriate test where the ratio of death rates between two treatment arms is not constant over time. The latter test places more weight on earlier as opposed to late patient events and time points. This approach may be more desirable when crossover is allowed or when patients are treated with second- or third-line agents.

## 2. INITIAL RESULTS

Between August 2004 and August 2005, 750 patients were accrued from 101 centers. The treatment arms were balanced with respect to baseline demographics and disease characteristics. With regard to ecog ps, 62% of patients had a ps of 0, and 38% had a ps of 1. With regard to the Memorial Sloan–Kettering Cancer Center risk classification, 38% had a favourable score, 56% had an intermediate score, and 6% were scored as poor risk.

Table i summarizes the published results to date, which report on pfs and response rates. Patients treated with sunitinib had a significantly longer pfs than did those treated with interferon: 11 months as compared with 5 months, which corresponds to a hazard ratio of 0.42 [95% confidence interval (ci): 0.32 to 0.54 months; *p* < 0.001]. Thus, in terms of pfs, the study met the primary endpoint. With respect to objective response rates, the initial rate reported with sunitinib was 31% as compared with 6% with interferon (*p* < 0.001). Health-related quality of life (qol) was reported to be superior with sunitinib. At asco 2007, the pfs data were also presented according to the Memorial Sloan–Kettering Cancer Center risk factors, such that for favourable-risk patients taking sunitinib, pfs was 14.5 months; for intermediate-risk patients, 10.6 months; and for poor-risk patients, 3.7 months, as compared with 7.9 months, 3.8 months, and 1.2 months for patients taking interferon 3.

After these results were first presented at asco 2006, sunitinib was very quickly declared the new standard of care in this patient population. Sunitinib did not receive Health Canada approval until August 2006, and it then took several months for the individual provincial bodies to decide whether to fund this agent. Sunitinib is now publicly funded in some way in all provinces.

## 3. UPDATED DATA

At asco 2008, the much-anticipated data related to os were presented orally (abstract 5024 4). despite the anticipation for this presentation, clinicians who had been treating metastatic rcc patients during the years leading up to June 2008 knew from experience that the addition of targeted therapies, and specifically sunitinib, into everyday practice prolonged the lives of patients with metastatic renal rcc.

Table i summarizes the updated pfs and response rate data. objective response rates, as determined by independent reviewers, were 39% with sunitinib and 8% with interferon alfa (*p* < 0.000001). Median pfs had not changed. of the study patients, 14% were still on sunitinib, and 2% were still on interferon.

The os with sunitinib was 26.4 months (95% ci: 23 to 32.9 months) as compared with 21.8 months (95% ci: 17.9 to 26.9 months) with interferon alfa. The log-rank p value was 0.051, and the Wilcoxon *p* value was 0.0128. Because the log-rank *p* value exceeded 0.05, some readers may interpret the study as negative; however, it is important to remember what a “*p* value” means. A *p* value of 0.05 represents a 5% likelihood of obtaining these results by chance alone. A *p* value of 0.051 represents a 5.1% likelihood of obtaining these results by chance alone.

Why was os not more dramatically improved? The fact that patients crossed over to sunitinib and also that many received second- or third-line therapy has to be taken into account. In the sunitinib arm, 56% of patients received post-study therapy, and in the interferon arm, 59% received post-study therapy as shown in Table ii. The influence of second-, third-, and even fourth-line treatment in metastatic rcc was never an issue before the use of targeted therapies, but it has now become a major confounder, as was seen in the target (Therapeutically Applicable Research to Generate Effective Treatments) study comparing sorafenib with placebo in the second-line setting 5. Crossover from the placebo arm to active sorafenib influenced survival, and the os benefit was subsequently not statistically significant. It is known from the target study that vegf-targeted therapy post interferon does prolong survival and thus would definitely have influenced the interferon-treated arm in the Motzer study.

Because of the influence of post-study treatment, the authors also presented the survival data in two other ways. The first was to censor the 25 patients from the interferon arm who crossed over to the sunitinib arm. The second was to exclude all patients who received any other post-study treatment. This left 193 patients in the sunitinib arm with a median os of 28.1 months (95% ci: 19.5 months to not reached), and 162 patients in the interferon arm with a median os of 14.1 months (95% ci: 9.7 to 21.1 months), log-rank *p* = 0.003. The hazard ratio in this analysis is 0.647 (95% ci: 0.483 to 0.870). These post hoc analyses obviously introduce bias, because there was likely a systemic difference in patients who did and who did not receive post-study treatment.

Another important component of the study was the evaluation of qol. This evaluation was conducted using several qol measures, including the Functional Assessment of Cancer Therapy–General (fact-G), the fact Kidney Symptom Index (fksi, a validated 15-question symptom index for kidney cancer patients) and disease-Related Symptoms subscale (a 9-item subscale of fksi that pertains more specifically to kidney cancer symptoms), the EuroQol-5D utility score, and the EuroQol visual analog scale for overall health state. This randomized controlled trial is the largest in metastatic rcc comparing patient qol in two different treatment arms, and the results from the qol analysis have recently been published 6. The primary endpoint focused on disease-related symptoms, not treatment-related adverse events; however, the overall health questionnaires should also have captured treatment-related adverse events. Patients on sunitinib have significantly better qol as compared with patients on interferon. The positive benefit is largely attributable to between-group differences (that is, sunitinib versus interferon), rather than to within-group differences (that is, improvement from baseline over time with sunitinib).

## 4. DISCUSSION

What is the bottom line?

Canadian medical oncologists are now seeing patients with metastatic rcc achieve median survivals of more than 2 years. These results are unprecedented. Sunitinib produces a pfs benefit over interferon alfa in patients with clear-cell metastatic rcc treated in the first-line setting. The primary endpoint was pfs, and the study was positive. Although the *p* value for os in the intent-to-treat analysis was 0.051, the reported difference in os is quite likely an underestimation of the true benefit, given the effect of post-study treatment. Such confounders will remain an ongoing problem in metastatic rcc trials now that several agents are active; the likely result is that pfs will be the major endpoint in future studies.

## Figures and Tables

**TABLE I:** Outcomes

	*Sunitinib*	*Interferon*	p *Value*
Patients (*n*)	374	337	
Objective response rates, independent review [% (95% confidence interval)] Initial report	31 (26–36)	6 (4–9)	0.001
Updated (asco 2008)	39 (34–44)	8 (6–12)	0.000001
Progression-free survival [months (95% confidence interval)] Initial report	11 (10–12)	5 (4–6)	0.001
Updated (asco 2008)	11 (11–13)	5 (4–6)	0.000001

asco= American Society of Clinical oncology (annual meeting).

**TABLE II:** Post-study therapy

	*Sunitinib* (*%*)	*Interferon* (*%*)	p *Value*
Any further therapy	56	59	ns
Sunitinib	11	33	ss
other vegf-targeted	33	32	ns
Cytokines	20	13	ns
Inhibitors of mtor	9	4	ns
Chemotherapy	6	6	ns

ns = nonsignificant; ss = statistically significant; vegf = vascular endothelial growth factor; mtor = mammalian target of rapamycin.
